# Supplementary material for: Mesoscale simulations predict the role of synergistic cerebellar plasticity during classical eyeblink conditioning
Source: PLoS Comput Biol. 2024 Apr 4;20(4):e1011277. doi: 10.1371/journal.pcbi.1011277 (PMC11060558; doi:10.1371/journal.pcbi.1011277)
Supplement: S1 Text — Number of elements in the olivocerebellar network (downbound and upbound neuron numbers in brackets), and model parameters for each neuron type, from [55]. Table B. Connectivity in the network. Connection types in the olivocerebellar network are reported together with the global average convergence and divergence ratios, and synaptic parameters, i.e. weight and delay. Excitatory and inhibitory connections are reported with positive and negative synaptic weight, respectively. When differentiated, downbound and upbound weights are reported (downbound; upbound). Equation A. pf-PC plasticity model. Learning rule for the pf-PC plasticity site, derived from previous works. (DOCX) [file pcbi.1011277.s009.docx]

# Supporting information

**Table A** ***Neurons in the network.***

| Neuron type | Number | C_m_  [pF] | τ_m_  [ms] | E_L_  [mV] | t_ref_  [ms] | V_r_  [mV] | V_th_  [mV] | k_adap_  [MH^-1^] | k_2_  [ms^-1^] | A_2_  [pA] | k_1_  [ms^-1^] | A_1_  [pA] | I_e_  [pA] |
| --- | --- | --- | --- | --- | --- | --- | --- | --- | --- | --- | --- | --- | --- |
| **GoC** | 70 | 145 | 44 | -62 | 2 | -75 | -55 | 0.217 | 0.023 | 178.01 | 0.031 | 259.988 | 16.214 |
| **GR** | 28615 | 7 | 24.15 | -62 | 1.5 | -70 | -41 | 0.022 | 0.041 | -0.94 | 0.311 | 0.01 | -0.888 |
| **MLI** | 446  (299 BC;  147 SC) | 14.6 | 9.125 | -68 | - | -78 | -53 | 2.025 | 1.096 | 5.863 | 1.887 | 5.953 | 3.711 |
| **PC** | 99 (69+30) | 334 | 47 | -59 | 0.5 | -69.0 | -43 | 1.491 | 0.041 | 172.622 | 0.195 | 157.622 | 742.534;  176.26 |
| **DCN_p_** | 133 (93+40) | 142 | 33 | -45 | 1.5 | -55 | -36 | 0.408 | 0.047 | 3.477 | 0.697 | 13.857 | 75.385 |
| **DCN_GABA_** | 78 (54+24) | 56 | 56 | -40 | 3.02 | -55 | -39 | 0.079 | 0.044 | 176.358 | 0.041 | 176.358 | 2.384 |
| **IO** | 13  (9+4) | 189 | 11 | -45 | 1 | -45 | -35 | 1.928 | 0.091 | 1358.197 | 0.191 | 1810.923 | -18.101 |
| **mf** | 117 | - | - | - | - | - | - | - | - | - | - | - | - |
| **gloms** | 2336 | - | - | - | - | - | - | - | - | - | - | - | - |

Number of elements in the olivocerebellar network (downbound and upbound neuron numbers in brackets), and model parameters for each neuron type, from [1].

**Table B *Connectivity in the network***

| **Connection type** | **Convergence** | **Divergence** | **Weight [nS]** | **Delay [ms]** |
| --- | --- | --- | --- | --- |
| *mf*-glom | 1 | 20 | 1 | 1 |
| glom-GrC | 4 | 49 | 0.23 | 1 |
| glom-GoC | 56.3 | 1.7 | 0.24 | 1 |
| GoC-GrC | 2.4 | 1000 | -0.24 | 2 |
| GoC-GoC | 16 | 16 | -0.007 | 4 |
| GrC(*aa*)-GoC | 320.4 | 0.8 | 0.82 | 2 |
| GrC(*aa*)-PC | 197.6 | 0.7 | 0.88 | 2 |
| GrC(*pf*)-GoC | 907.9 | 2.2 | 0.05 | 5 |
| GrC(*pf*)-PC | 1463 | 5 | 0.14 | 5 |
| GrC(*pf*)-SC | 480 | 5.1 | 0.18 | 5 |
| GrC(*pf*)-BC | 740 | 3.8 | 0.1 | 5 |
| BC-PC | 20 | 14 | -0.44 | 4 |
| SC-PC | 5.4 | 1.8 | -1.64 | 5 |
| BC-BC | 14 | 14 | -0.006 | 4 |
| SC-SC | 14 | 14 | -0.005 | 4 |
| *mf-*DCNp | 48 | 1 | 0.35 | 4 |
| PC-DCNp | 33 | 45 | -0.6; -0.3 | 4 |
| PC-DCN_GABA_ | 14 | 12 | -0.04; -0.07 | 4 |
| IO-PC | 1 | 6 | 300 | 4 |
| IO-SC | 2 | 41 | 2.5 | 40 |
| IO-BC | 3 | 41 | 2.5 | 40 |
| IO-DCNp | 6 | 62 | 2.5 | 4 |
| IO*-*DCN_GABA_ | 6 | 38 | 0.1 | 5 |
| DCN_GABA_*-*IO | 38 | 6 | 0.75 | 25 |
|  |  |  |  |  |

Connection types in the olivocerebellar network are reported together with the global average convergence and divergence ratios, and synaptic parameters, i.e. weight and delay. Excitatory and inhibitory connections are reported with positive and negative synaptic weight, respectively. When differentiated, downbound and upbound weights are reported (downbound; upbound).

**Equation A. *pf-PC plasticity model***

The *pf-PC* plasticity rule was derived from previous works [34,36], and follows the equations:

$${\Delta W}_{{pf}_{i}{\to PC}_{j}}\left( t \right)=\left\{ \begin{aligned} {-LTD}_{PC} \int_{-\infty}^{t_{{cf\_spike}_{j}}} K\left( t_{{cf\_spike}_{j}}-x \right)\delta_{{pf}_{i}}\left( t_{{cf\_spike}_{j}}-x \right)dx, if {pf}_{i} is active and t= t_{{cf\_spike}_{j}} \\ {LTP}_{PC}, if {pf}_{i} is active and t\neq t_{{cf\_spike}_{j}} \\ 0, otherwise \end{aligned} \right.$$

Where:

$$\delta_{{pf}_{i}}\left( t \right)= \left\{ \begin{aligned} 1, if {pf}_{i} is active at time t \\ 0, otherwise \end{aligned} \right.$$

And the Kernel function is:

$$K\left( t \right)= A{\cdot( e}^{-( \frac{t-t0}{\tau})} )\cdot{(sin(2\pi\frac{t-t0}{\tau})}^{20}$$

Where $t0$ was set to 100 ms based on physiological delays of the cerebellar circuit plasticity, and $A$ and $\tau$ were set to normalize the kernel.

# References

[1] Geminiani A, Casellato C, D’Angelo E, Pedrocchi A. Complex electroresponsive dynamics in olivocerebellar neurons represented with extended-generalized leaky integrate and fire models. Front Comput Neurosci. 2019;13. doi:10.3389/fncom.2019.00035
