# Supplementary material for: Mesoscale simulations predict the role of synergistic cerebellar plasticity during classical eyeblink conditioning
Source: PLoS Comput Biol. 2024 Apr 4;20(4):e1011277. doi: 10.1371/journal.pcbi.1011277 (PMC11060558; doi:10.1371/journal.pcbi.1011277)
Supplement: Attachment — Submitted filename: answer_reviewers.pdf [file pcbi.1011277.s010.pdf]

We thank the Editors and Reviewer for their thoughtful and detailed comments, which significantly helped to improve the results and the manuscript clarity. We are now submitting a modified version, where we have added results from simulations proving the robustness of the model, discussion on some further neural mechanisms that contribute to CEBC and could be included in future implementations of the model, clarified methods on model construction and parameters.
Please, find details below together with corresponding parts of the manuscript that were updated, in track changes.

Reviewer #1:

CEBC has been well-studied by many researchers, including Drs. Alkon, Mauk, Thompson, de Zeeuw, and Hesslow. While there are agreement and disagreement among research groups (e.g., if synaptic plasticity is involved in the learning), this manuscript answers an important question of how the synergy of MLI inhibitory synaptic transmission and excitatory synaptic plasticity is involved in the CEBC learning process. The author built the in silico cerebellar system using a supercomputer with a high temporal resolution and challenged the motor learning theory. The conclusion was plausible in their relatively simplified system, and the resultant message closely aligned with previous experimental outcome from coauthors of Prof. de Zeeuw's group (BoeleH De ZeeuwCI 2018sciadv).

My questions and comments are following:

1.
I wonder the intrinsic excitability of neurons is a critical factor, which may change the simulation outcome. I would like to ask the result as an independent figure when PC excitability is set high (e.g., basal firing frequency rate to 150% increase) and low (e.g., basal firing rate to 60% decrease) in both upbound and downbound PCs.

We run two additional simulations with PCs basal firing rate decreased (to 50%) and increased (to 150%). In both cases, learning was reduced/slowed-down (Fig. S_B), demonstrating the importance of physiological values of PC spontaneous firing, as better detailed in point 3. below.

"

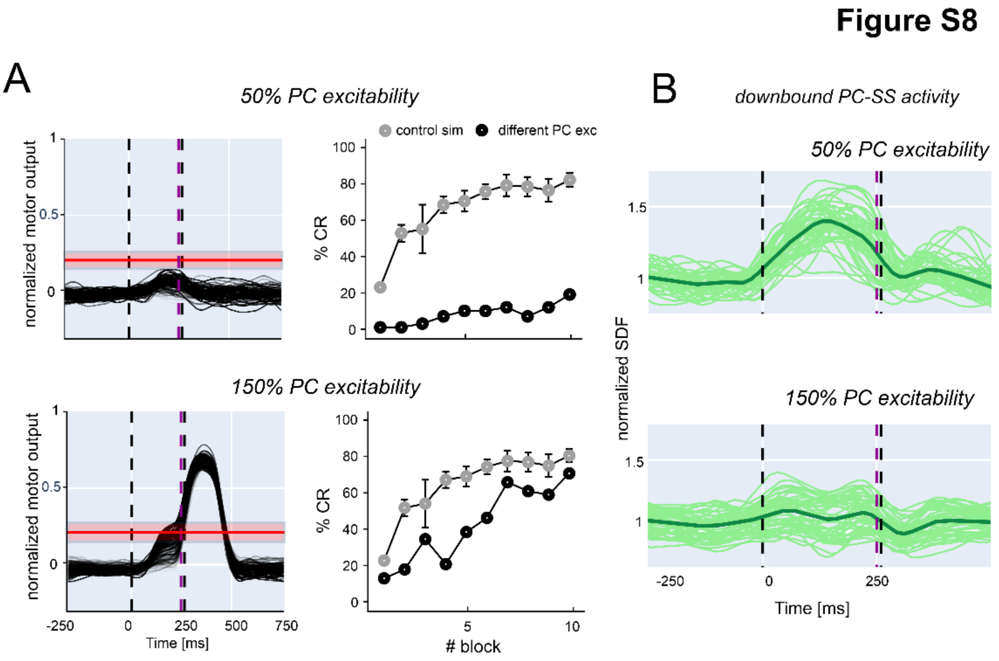

S8 Fig. Simulations with altered intrinsic excitability of PCs. (A) Motor output with decreasing (top) and increasing (bottom) PC spontaneous firing: both the motor response and the learning curve are

reduced or slowed down. (B) A different pattern of facilitation and suppression at the end of learning is observed in downbound PCs, where the unbalance between the two mechanisms causes an insufficient release of the DCN and thus the observed alteration of learning."

2.
Schreurs and Alken (1991; 1997)(Schreurs et al., Brain Res. 1991 doi: 10.1016/0006-8993(91)91100-f. PMID: 1868333; Schreurs et al., J Neurophysiol. 1997 doi: 10.1152/jn.1997.77.1.86. PMID: 9120599) suggested an increase in the PC firing frequency during CEBC and excitability of dendrites. However, the manuscript merely discuss:
"655 The neurons were single-point but maintained salient discharge properties, e.g., non-linear I-f
656 relationship, burst/pause and rebound responses [56]. The absence of dendritic integration is a minor
657 issue, since the CEBC input is "mono-dimensional" (a tone) and does not require dendritic integration
658 over an extended multiparametric space. …
I consider PC dendrites more complex than the authors expect, and they have both dendritic integration, branch-specific compartmentalization, and branch-specific enhancement (Zang et al./De Schutter 2018 Cell Rep; Ohtsuki et al./ Hansel, 2012 Neuron; Busch & Hansel, 2023 Science). Therefore, the claim of this manuscript may miss a biological significance. One of the researchers found that the difference of the branch excitability confers the branch-specific information processing of synaptic transmission, too (Ohtsuki, 2020 JNS). I wish the authors to describe the comment on it with referring citations as a paragraph. In fact, L7-SK2 mice show impaired CEBC (Grasselli, Boele, / Hansel 2020 PLoS Biol). Thus, this manuscript will be established if it append the discussion as for another hypothesis or prediction. L.655-658 should be rephrased.

Zang Y, Dieudonné S, De Schutter E. Cell Rep. 2018;24(6):1536-1549. doi: 10.1016/j.celrep.2018.07.011. PMID: 30089264
Ohtsuki G, Piochon C, Adelman JP, Hansel C. Neuron. 2012;75(1):108-20. doi: 10.1016/j.neuron.2012.05.025. PMID: 22794265
Busch SE, Hansel C. Science. 2023;381(6656):420-427. doi: 10.1126/science.adi1024. PMID: 37499000
Ohtsuki G. J Neurosci. 2020;40(2):267-282. doi: 10.1523/JNEUROSCI.3211-18.2019. PMID: 31754008
Grasselli G, Boele HJ, Titley HK, Bradford N, van Beers L, Jay L, et al. PLoS Biol. 2020;18(1):e3000596. doi: 10.1371/journal.pbio.3000596. PMID: 31905212

We agree with the reviewer that dendritic computation and variations in dendritic excitability are key features of Purkinje cell processing and lines 655-658 could be misleading. In the current work, we choose to represent single neurons (including PCs), as point neurons as a compromise between biological plausibility and computational load. Indeed, in CEBC, the input is a monodimensional signal (a LED light/tone) that does not vary in time. According to the canonical cerebellar circuit theory where pattern separation occurs in the granular layer and integration happens at the Purkinje cell level, PC dendritic integration in CEBC is less crucial than in more complex tasks where the cerebellum receives multi-modal and time-varying sensorimotor inputs. In addition, here we wanted to focus on synaptic plasticity mechanisms that modify PC output, even if we are aware that also dynamic dendritic mechanisms may play a role in the modifications of PC activity driving learning. Reducing neurons to point neuron models also allowed us to limit the computational load of simulations and thus simulate a large-scale neural network with distributed plasticity, which are both computationally expensive. We have added a better explanation of this in the Methods:

" The network was simulated as a spiking neural network made of point neurons, modelled as ~~The SNN neurons were~~ Extended-Generalized Leaky Integrate and Fire (E-GLIF). This model allows to keep the main electroresponsive features of cerebellar neurons, while reducing the computational load of simulations [56], making it feasible to simulate a large-scale SNN with realistic population sizes

and millions of plastic synapses, even if requiring high-performance computing resources (see Par. 2.4)."

And a full paragraph in the Discussion:

"The large number of trials required to simulate plastic changes during CEBC learning in mice and the jump to mesoscale (cerebellar cortex associated with DCN and IO embedded in a controller) required some simplifying assumptions. ~~which, n~~Nonetheless, ~~had~~ these were purposefully designed to keep ~~limited impact on~~ the biological plausibility of ~~these~~ simulations, considering the task and the mechanisms within the micromodules under investigation.

The neurons were single-point but maintained salient discharge properties, e.g., ~~non-~~linear I-f relationship, burst/pause and rebound responses [57]. ~~The absence of dendritic integration is a minor issue, s~~Since the CEBC input is "mono-dimensional" (a tone/LED light) and not time-varying, sparse and non-recurrent encoding in the granular layer was used to discriminate the time instants of the CS at the Purkinje layer level [10,11,50,61,76], in absence of dendritic integration.

Recent studies elucidated the role of PC dendritic processing in cerebellar computation and learning: differentiated excitability of PC dendritic tree makes active inputs heterogeneous and contribute to pattern separation, and dendritic excitability can be plastic [77–79]. This is crucial also for PC responses to *cf* inputs that are the instructive signal for CEBC: clustered activation of the dendritic tree from *pf* and MLI inputs causes distinct responses to *cfs* [80]. This can involve multiple *cf* innervations of the PC dendritic tree especially in the cerebellum of human and higher non-human primates [81]. Differentiated excitability and plasticity of PC dendrites could be an additional mechanism contributing to CEBC, together with synaptic plasticity, as shown in [82] where a lack of dendritic excitability leads to reduced CEBC. This could be investigated in future implementations of the current SNN model, where PCs are represented as multi-compartmental neurons with dendritic processing instead of point neurons. Adding dendritic computation will be crucial to simulate more complex tasks, where the cerebellum receives multi-modal and time-varying inputs."

3.
Related to the question above, Prof. Sang Jeong Kim's group recently found the LTD of intrinsic excitability in animals (Shim et al. /Kim 2017JNS doi:10.1523/JNEUROSCI.3464-16.2017 pmid:28495974), which potentially reduces PC firing frequency without inhibitory conductance. I wish the authors to ask the possibility of the plasticity in their model as discussion.

We agree that plasticity of intrinsic excitability may play an important role in controlling PC pauses. We have discussed this more explicitly together with further citations.

"Our study focused on synaptic plasticity for CEBC in the cerebellar cortex, but additional plasticity mechanisms could contribute to the conditioned response learning. Plasticity of neuron intrinsic excitability has been shown recently to play a role in CEBC. Specifically, PC pauses can be reinforced by intrinsic electroresponsive mechanisms [72] and intrinsic plasticity, which can reduce spontaneous PC firing even without increased inhibitory input [8,94,95]. In addition, intrinsic excitability of dendrites in MLIs could contribute to increased MLI feedforward inhibition [96]. Preliminary simulations with altered intrinsic excitability show decreased or slowed-down CEBC (Fig. S8), due to an unbalance of facilitation and suppression mainly in downbound PCs: when PC baseline activity is lower, they are more sensitive to synaptic potentiation, resulting in facilitation of their activity at the end of learning [26], which prevents proper CEBC; when baseline activity is higher, depression still occurs but it takes longer to release the DCN, leading to slowed-down CEBC. Despite these promising preliminary results, the role of intrinsic plasticity mechanisms contributing to conditioned response timing remains to be evaluated [89–91] and should be considered in future implementations of the model."

4.
In the model, I wonder about the conductance of MLI GABAergic projection. How many projections of stellate cells and basket cells are assumed? And how much conductance is given in the authors' model? I ask the authors to provide every parameter of synaptic conductance and basic property of each

neuron, as an additional table.
Given LTP of pf-MLI synapse or LTP of MLI intrinsic excitability was induced, is the +321%+/-325 facilitation (Table 1) plausible? I've never heard of it in vivo and in vitro, to my knowledge. Some more complementary mechanisms may be inherited (cf. a synergy of intrinsic excitability of dendrites and synaptic transmission Zhang and Linden, 2003 https://doi.org/10.1038/nrn1248).

All connectivity parameters (convergence/divergence, synaptic weights and delays) are reported in Table S2 in the Supplementary material. We also added single neuron parameters in Table S1 that originally reported only the number of neurons/units in the network.

Regarding the MLI GABAergic projection, it was differentiated for stellate and basket cells (average divergence from stellate and basket to Purkinje cells equal to 1.8 and 14, respectively); this was obtained from neuron morphology intersection algorithms as detailed in De Schepper et al., Comms Bio, 2022 (https://doi.org/10.1038/s42003-022-04213-y).

MLI facilitation was driven by *pf*-MLI LTP. The values reported in Table 1 are from Ten Brinke et al., 2015 (Table S2); even if the amount of facilitation is significantly high, the reached firing rate is within physiological ranges for MLIs.

We agree that further mechanisms could contribute to MLI facilitation besides synaptic plasticity of connections from parallel fibers; we addressed this in the Discussion (added paragraph for comment 2. above).

5.
Data and Code Availability

https://github.com/AliceGem/cereb_scaffold_ebc does not work.

The link was updated to https://github.com/AliceGem/mesoscale_simulations_cebc .

6.
l. 124-126 was not understandable for me.

124 Furthermore, complex spikes were extracted from PC in vivo recordings: throughout learning, a
125 complex spike response emerged during the ISI (conditioned complex spike), with 43% probability
126 of occurrence in fully trained mice, at an average latency of 88 ms after CS onset.

This was clarified:

"Furthermore, complex spikes were extracted from PC *in vivo* recordings.~~:~~ This analysis showed that throughout learning, a complex spike ~~response~~ emerged during the ISI (conditioned complex spike), with 43% probability of occurrence in fully trained mice, at an average latency of 88 ms after CS onset. To date, the mechanism causing this complex spike response (and thus IO/cf activity that is the source of complex spikes) remains unknown, but it may well originate from extra-cerebellar regions [47]."

7. Abbreviation.

l. 246, Spike Density Function

l. 312, spike density function (SDF)

This was fixed.

8.
Correct? Which one?
l. 352-353
see also Fig. 4 below

The references to figures were better specified:

"[…] CEBC simulations matched the physiological parameter range measured in mice [19,25,27,64,65] concerning basal firing rate and firing rate modulation after learning (Fig. S3B, S5, Table 1), and CR learning rate and timing in control conditions (Fig. S3B, S5, Table 1, see also (Fig. 4 below)."

9.
Figure 4C
What do asterisks mean by?

Asterisks mark significantly different distributions; we added this information in the caption.

10.
l.573-582
Description on Fig.7 is missing.

Fig. 7 caption is in lines 628-633 of the updated manuscript.

Editorial review

This paper extends previous work on large-scale spiking models of cerebellar circuits in the context of classical eyeblink conditioning (CEBC). The key new features are the inclusion of two cerebellar modules with different properties and plasticity in the feedforward inhibitory loop, in addition to the parallel fibre to Purkinje cell plasticity that has been the focus of most previous models. The simulation demonstrates that these plasticity mechanisms interact such that only their combined absence has a significant effect on learning, consistent with experimental results from mice using mutations that target one or other or both systems.

Overall this is an interesting study that provides insight from an anatomically and physiologically constrained model into cerebellar function. This includes reproducing the genetic knockout effects and providing some predictions of the expected changes in microcircuits during learning, i.e., which cells should show significant activity changes during learning. There is an interesting discussion of the insights provided by the model as well as additional factors that might be relevant to include in future modelling.

However a number of revisions are needed **to improve the clarity of the paper**.

Line 26 "prompts to remap" could be better expressed.

The sentence was rephrased:

"However, recent experimental evidence the discovery of multiple forms of plasticity distributed over different cerebellar circuit synapses prompts to remap challenges this relatively monopolistic view of the cerebellar learning sites."

27-28 first mention of 'upbound' and 'downbound' modules. For a reader not highly familiar with the cerebellar system it remains rather unclear throughout most of the paper what these terms refer to, so some explanation is needed earlier.

The description of upbound and downbound modules was added in the abstract:

"However, recent experimental evidence the discovery of multiple forms of plasticity distributed over different cerebellar circuit synapses prompts to remap challenges this relatively monopolistic view of the cerebellar learning sites. Bidirectional plasticity appears crucial for learning, in which different microzones can undergo opposite changes of synaptic strength (e.g. downbound microzones – more likely depression, upbound microzones - more likely potentiation), and multiple forms of plasticity have been identified, distributed over different cerebellar circuit synapses."

And improved in the introduction, to avoid any ambiguity throughout the paper:

"[…]. This suggests that multiple cerebellar microzones and modules receiving at least in part different input signals may be involved in CEBC: downbound modules, including zebrin-negative (Z-) microzones, where PCs are more likely to undergo SS suppression, and upbound modules, potentially including zebrin-positive (Z+) microzones, where PCs are more likely to undergo SS facilitation."

38-39 "fine tuning of adaptive associative behaviours at a high spatio-temporal resolution" - this indeed seems like it should be the most interesting outcome of the presented work, in particular that this might explain why there are multiple forms of plasticity in a circuit that ultimately is learning a rather simple task. However, it is not sufficiently demonstrated in this paper that the circuit performs 'fine tuning', e.g., there is no exploration of how the circuit performs when details of training, such as ISI, are varied.

We have added the results of 2 additional simulations with different ISIs (200ms and 300ms, shorter and longer than the one used originally – 250ms), to show how the model perform fine-tuning of motor response timing. In both cases, a physiological learning curve with gradually increasing %CR was generated. However, in each condition, the timing of the motor response was adjusted to anticipate the different US timing (Fig. S_A), proving that the model can generalize to different stimuli conditions.

In the Results:

"In control, the simulated CEBC learning curve attained 81% CR (last block) following a bounded exponential curve, closely matching the experimental one measured in mice [19] (Fig. 4A). Similar motor output and learning curves were obtained for simulations with shorter (200 ms) and longer (300 ms) ISI durations, proving the robustness of the model in fine-tuning the timing of the motor output (Fig. S4)."

As Supplementary figure:

"

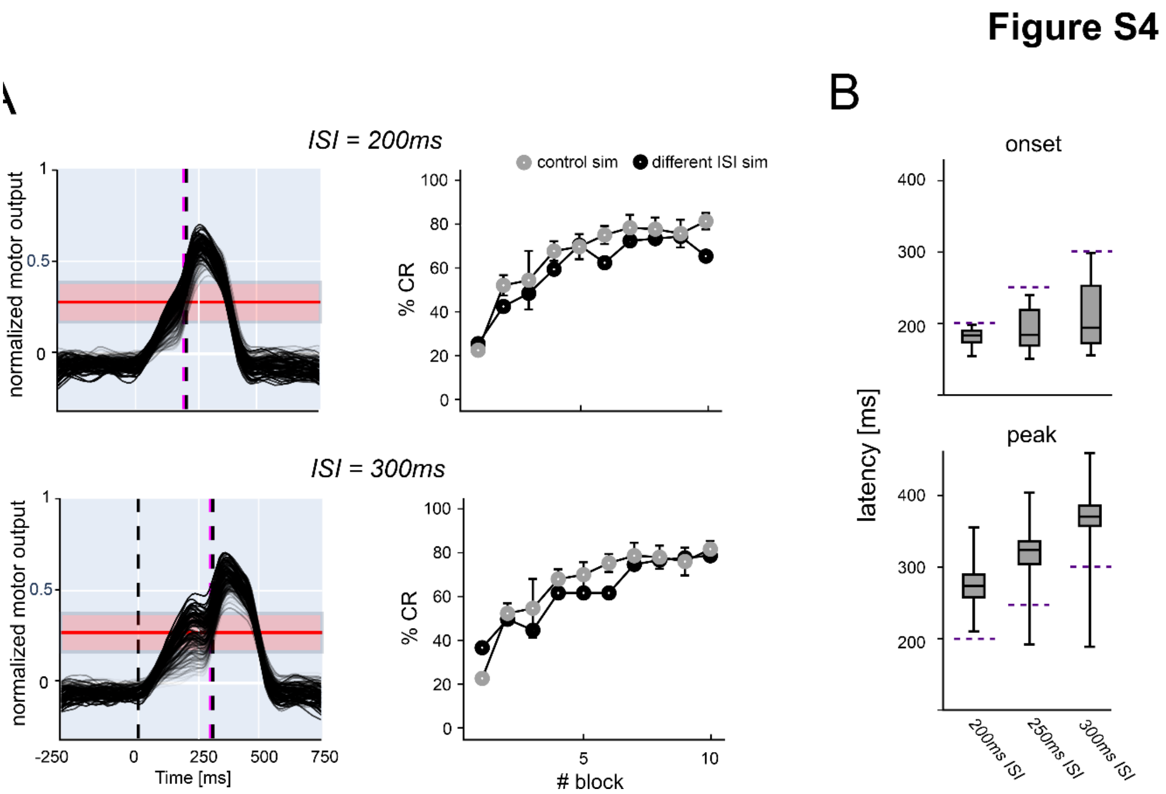

S4 Fig. Simulations with different ISIs. (A) Motor output and %CR in the 200 ms (top) and 300 ms (bottom) ISI simulations. Both motor output evolution and %CR increase throughout learning blocks,

as in the simulations with ISI=250 ms. **(B)** Onset and peak timing of the conditioned response. The motor output timing is adjusted to the US, which occurs at different ISIs in the different simulation conditions."

45 It seems odd to emphasise here the co-termination of the CS and US as the defining feature of the CEBC paradigm, whereas the critical factor for generating a "well-timed" CR is the temporal relationship between the onset of the CS and US, not their termination.

We had specified the cotermination of CS and US, to highlight that we refer to delay and not trace eyeblink conditioning (where CS terminates before US starts). However, the description was misleading and not complete; we have rephrased it to stress the key elements of CEBC:

"In this simple task, two sensory stimuli are provided in sequence ~~and co-terminate~~ time-locked to each other: ~~; these include~~ a neutral conditioned stimulus (CS, usually a tone or a LED light) followed at a specific Inter-Stimulus Interval (ISI) by an unconditioned stimulus (US, usually an air puff or a periorbital electrical stimulation), with the CS and US co-terminating at the same moment at the end. After repeated CS-US presentations with a constant ISI, the cerebellum generates a well-timed conditioned response (CR, i.e., eyelid closure) that anticipates the US."

63-66 I found this unclear, why would PC firing be reduced by an increase in the pf input? Isn't the hypothesis that pf-PC LTD decreases the pf input, and hence the PC firing? And in what sense can PC firing "hardly be reduced" by a change in pf input?

The sentence contained a mistake ("increase" instead of "decrease"). It was corrected and rephrased:

"Second, in absence of any synaptic input PCs exhibit ~~the~~ high-frequency ~~spontaneous~~ intrinsic firing, which presumably ~~of PCs~~ can hardly be reduced by a~~n~~ ~~increase~~ decrease in the activity of its direct *pf* excitatory input [16,17]. ~~,~~ This suggests that ~~so that~~ *pf*-PC LTD by itself cannot be sufficient for the SS suppression of PCs during CEBC [18,19], ~~. Instead, PC-SS suppression is largely driven by~~ and ~~instead~~ that synaptic inhibition [20–22] from molecular layer interneurons (MLIs) is likely to play a major role in driving PC-SS suppression [19,23,24]."

70-72 why does the role of pf-PC LTD "to prevent potentiation" not suffice to bring SS activity of PC down?

The fact that PCs exhibit spontaneous firing suggests that an active inhibitory mechanism should be needed to bring SS activity down. The whole sentence was rephrased (previous point).

78-80 Again to a reader unfamiliar with the cerebellum, it is entirely opaque what is being referred to for "zebrin-negative" and "zebrin-positive"

A more detailed explanation of upbound and downbound modules and their relationship to Z+ and Z- PCs was added:

"Finally, while ~~While downbound microzones appear to be responsible for the~~ PC-SS suppression ~~that~~ is critical for expression of the conditioned responses (CRs) of the eyeblinks [26], SS facilitation ~~adjacent upbound microzones~~ may also contribute, to some extent, to spatiotemporal control of the CRs [19,27]. This suggests that multiple cerebellar microzones and modules receiving at least in part different input signals may be involved in CEBC: downbound modules, including zebrin-negative (Z-) microzones, where PCs are more likely to undergo SS suppression, and upbound modules, potentially including zebrin-positive (Z+) microzones, where PCs are more likely to undergo SS facilitation."

140-144 This seems a very brief explanation of the modelling approach, is it sufficiently covered in the cited references?

This paragraph was specifically referring to software used for model building and simulation. We have reorganized this part of the methods to first explain all the steps of the modelling approach in detail and then the software.

147-162 this seems like material that belongs in the introduction.

The first part was moved to introduction (until line 153). The lines were left as an introductory paragraph in Methods, as they refer to the model used in the current work.

170-193 This description of the model has insufficient detail. E.g. it provides the total number of cells but not the number of each cell type, for ii) and iii) not even the number of cells. Just listing the connection types seems inadequate.

Quantitative info on network architecture (e.g. number of neurons per cell type, conv/div of connections etc) is reported in Tables S1 and S2 in supplementary materials, which were wrongly referenced. We have now fixed the reference in Par. 2.2.1, and added a more exhaustive explanation of network connectivity:

"In the cerebellar cortex, input signals from *mf* are transmitted to the granular layer via excitatory connections through the glomeruli (*mf*-glom, glom-GrC, glom-GoC). The granular layer includes recurrent excitatory (from GrC) and inhibitory (from GoC) connections (GoC-GrC, GoC-GoC, GoC-GoC gap junctions, GrC(*aa*)-GoC, GrC(*pf*)-GoC). Signals are then transmitted to Purkinje and Molecular layers through *aa* and *pf* projections (GrC(*aa*)-PC, GrC(*pf*)-PC, GrC(*pf*)-SC, GrC(*pf*)-BC). In turn, MLIs inhibit both PCs and other MLIs (SC-PC, BC-PC, SC-SC, BC-BC connections). In the DCN, DCN$_{p}$ neurons receive excitation from *mf* (*mf*-DCN$_{p}$) and IO (IO-DCN$_{p}$), and inhibition from PCs (PC-DCN$_{p}$), while DCN$_{GABA}$ neurons are inhibited by PCs (PC-DCN$_{GABA}$) and excited by IO collaterals (IO-DCN$_{GABA}$). IO neurons transmit excitatory signals to PCs, causing complex spikes (IO-PC), and to MLIs through spillover connections (IO-SC and IO-BC). IO neurons also receive feedback inhibition from DCN through DCN$_{GABA}$-IO connections [55]. In our model, all connections involving PC, DCN and IO neurons are confined within a particular micromodule. Moreover, MLIs, the axons of which traverse predominantly in the sagittal plane [26], are also associated with upbound and downbound microzones based on whether they inhibit more upbound or downbound PCs, respectively. In summary, ~~T~~the cerebellar cortex model contains 16 connection types ~~(identified by their source and target neuronal population): *mf*-glom, glom-GrC, glom-GoC, GoC-GrC, GoC-GoC, GoC-GoC gap junctions, GrC(*aa*)-GoC, GrC(*aa*)-PC, GrC(*pf*)-GoC, GrC(*pf*)-PC, GrC(*pf*)-SC, GrC(*pf*)-BC, SC-PC, BC-PC, SC-SC, BC-BC,~~ [42], and~~.~~ t~~T~~he extension to the deep cerebellar and olivary nuclei brought out 9 more connection types.~~: *mf*-DCN$_{p}$ (excitatory), PC-DCN$_{p}$ (inhibitory), PC-DCN$_{GABA}$ (inhibitory), IO-PC (excitatory), IO-SC (excitatory), IO-BC (excitatory), IO-DCN$_{p}$ (excitatory), IO-DCN$_{GABA}$ (excitatory), DCN$_{GABA}$-IO (inhibitory).~~ The detailed network architecture information is reported in Supplementary material (Table S1 for neurons, Table S2 for connectivity)."

203 – what are 'parrot' neurons?

They are neurons that transmit the same spike train they receive. The term is specific to the NEST simulator nomenclature; we have substituted it with 'relay units' and explaned:

"Glomeruli and mossy fibers were modelled as relay units, transmitting the same spike trains that they receive.~~ using NEST "parrot" neurons~~."

207 describes the parameters as being 'set' to reproduce physiological data. How was this done? Extracted directly from data? Hand tuning? Parameter search? Some automated fitting method?

Synaptic weights were set using a bisection search, initialized to the values in Geminiani et al., Front Comp Neurosci, 2019 (https://doi.org/10.3389/fncom.2019.00068).

We have specified:

"Synaptic weights, i.e., the synaptic conductance changes induced by presynaptic spikes, were tuned through bisection search initialized with the values in [43], ~~set~~ to obtain the physiological basal discharges in each neuronal population as ~~that was~~ recorded in behaving mice [19,22,27,58,59].".

Lines 216-239 The learning rule needs clearer explanation. It is described as STDP, but STDP is normally understood to refer to change in a synapse due to the relative timing of spikes in the presynaptic and postsynaptic neurons, with respect to that synapse. However here the change in pf-PC or pf-MLI (so pf is the presynaptic neuron and PC/MLI the postsynaptic neuron) depends on the relative timing of the spike in cf and pf. The text does not mention any dependence on MLI activity, yet in the equations this is a condition on the occurrence of LTP or LTD. It might also help in the equations to use 'cf' rather than 'IO' (or to refer to IO activity rather than cf activity in the preceding text) to make the connection between these terms explicit.

After reading further it is mentioned on line 385-386 (fig 1 caption) that the IO activity in cfs elicits a complex spike in PC and activates MLI; so I am inferring that the assumed mechanism here is that cf spikes cause (complex spike?) activity in PC or MLI, and this is the 'postsynaptic' activity that makes it an STDP learning rule? But the implemented equations use the cf spike, not the MLI spike, for the timing.

We used the term "STDP" based on the definition in previous work that developed the *pf*-PC learning rule (Luque et al., 2016), but we agree that it can be misleading, so we have clarified that it is spike-driven (because it depends on spikes) and supervised by *cf* activity (removing IO for clarity). The reference to MLI spikes was wrong, we corrected it accordingly. Indeed, the teaching signal is the *cf* spikes: when there is a *cf* spike, LTP occurs on the connections between active/previously active *pf* and the MLI that receive the *cf* teaching signal. When only *pf* are active without teaching signal, LTD occurs.

"In order to simulate learning neural mechanisms [60], the network model included long-term plasticity at the *pf-PC* and *pf*-MLI synapses. The learning rules implemented bidirectional spike-driven~~Spike-Timing Dependent P~~ plasticity supervised by the *cf*s.

The *pf-PC* plasticity rule was derived from previous works [34,36], based on the observation that *pf* stimulation coupled with *cf* activation (teaching signal) triggers ~~*pf*-PC~~ Long-Term Depression (LTD) at synapses between *pfs* and PCs receiving the *cf* signal, while *pf* stimulation alone causes *pf*-PC Long-term Potentiation (LTP) [13] (see also Supplementary material).

The *pf-MLI* plasticity rule was defined *de-novo* and implemented in NEST. The *pf*-MLI plasticity was constructed following the same principle of *pf-PC* plasticity, i.e., using a mechanism based on co-activation of *pfs* and *cfs,* but the sign of changes was reversed: a *cf* spike (teaching signal) causes LTP at *pf*-MLI synapses ~~that~~ between *pfs* that are active just before *cf* activation and MLIs receiving the *cf* signal. The LTP amount depends on the convolution of *pf* spikes with a kernel function."

For the sake of completeness, we have also added the *pf*-PC learning rule from previous studies in supplementary material.

241 Again please explain how the parameters were 'set'.

We tuned plasticity parameters using trial and error. We specified in the text:

"They were tuned using trial-and-error ~~set~~ in order to maximize the match between the experimental and the simulated firing modulation at the end of a short CEBC sequence (20 trials), for MLI, PC and DCN_p populations in the downbound microzone. […]"

262-266 This is not at all well described, but the impression provided is that the CIO was manually manipulated to change across training trials, in a way that mimics learning, but without this actually being an emergent property of the circuit or the use of any actual learning rule used to induce the change. It was not clear how the 'complex spike' fits into the overall system but it seems to be key later on (e.g. line 559) to explain residual learning after other factors are removed. On lines 425-428 it is described as a 'result' of the model that the "complex spike modulation during learning showed a typical evolution" but it seems this feature was directly built into the model. Please explain this better.

The origin of the conditioned IO/complex spike response is still debated and may be extra-cerebellar (Ten Brinke and De Zeeuw, Neurosci Letters, 2017, doi: 10.1016/j.neulet.2018.04.035). Therefore, we provided it as an external input to the cerebellar SNN, with parameters (latency and probability of occurrence) derived from experimental data. We let this input drive the network dynamics and the motor response as the other inputs, without any constraint. We have better explained this and rephrased to clarify that while the CIO is provided as an input based on experimental data, the emerging effects on network activity and behavior are unconstrained and prove sufficient to explain residual learning.

- "Conditioned IO response (CIO): a 400-Hz burst was delivered to half of IO neurons in the downbound microcomplex, with latency 88 ms after CS onset [19], causing a conditioned complex spike in PCs through *cf*. The occurrence probability of this input was linearly increased with the number of trials raising up to 43% at the end of learning. Given that the mechanistic origin of the CIO is still unknown and could potentially be extra-cerebellar [47], this input in the model was set based on, as observed experimental observations, and the parameters, e.g. probability of occurrence and latency, were extracted from Ten Brinke and colleagues ly [19]. The effect of the CIO on the whole network activity and behaviour was unconstrained and followed the mechanisms embedded in the SNN."

The sentence "complex spike modulation during learning showed a typical evolution" was misleading and was deleted.

409- 412 I missed anything in the earlier model description explaining this differential innervation of microzones by the MLI.

We have added more details in the description of connections:

"Moreover, MLIs, the axons of which traverse predominantly in the sagittal plane [26], are also associated with upbound and downbound microzones based on whether they inhibit more upbound or downbound PCs, respectively."

601 "predicting the impact of genetic mutations" - given these were known, and a focus in model construction, it seems too strong to say the model predicted these effects, rather, it was consistent with these observations.

The sentence was rephrased to clarify that while genetic mutations and the impact on behavior were known, their impact on neural and plasticity distribution was predicted by the model:

"These mechanisms were accounted for by a multiscale computational model, which was first validated against experimental data. KO simulations reproduced CEBC behaviour consistent with experiments from mice where genetic mutations conjunctively affected MLI feedforward inhibition and *pf*-PC LTD [25]. and tThen, the model was used to resolve the mechanistic link between microscopic neural circuit phenomena and sensorimotor learning in the cerebellum, explaining the impact of these genetic mutations conjunctively affecting MLI feedforward inhibition and *pf*-PC LTDon the underlying cerebellar neural mechanisms and predicting the behavioural outcome."
